# Effects of Co-Ingestion of β-Hydroxy-β-Methylbutyrate and L-Arginine α-Ketoglutarate on Jump Performance in Young Track and Field Athletes

**DOI:** 10.3390/nu13041064

**Published:** 2021-03-25

**Authors:** Piotr Kaczka, Katarzyna Kubicka, Amit Batra, Marcin Maciejczyk, Edyta Kopera, Justyna Bira, Tomasz Zając

**Affiliations:** 1 Academy of Physical Education in Katowice, Mikołowska 72a, 40-065 Katowice, Poland; kz.kubicka@gmail.com (K.K.); amit@op.pl (A.B.); tulipanka.e@gmail.com (E.K.); j.bira@interia.pl (J.B.); pbcz@awf.katowice.pl (T.Z.); 2 Department of Physiology and Biochemistry, Faculty of Physical Education and Sport, University of Physical Education in Krakow, Jana Pawła II 78, 31-571 Kraków, Poland; marcin.maciejczyk@awf.krakow.pl

**Keywords:** medium distance runners, β-hydroxy-β-methylbutyrate, HMB, Ca-HMB (calcium salt of HMB), L-Arginine α-ketoglutarate, AAKG, Arg, countermovement jump, creatine kinase, lactate dehydrogenase

## Abstract

The aim of the study was to determine the effect of simultaneous supplementation of β-hydroxy-β-methylbutyrate and L-Arginine α-ketoglutarate on lower limb power and muscle damage in medium distance runners aged 15.3 (±0.9) years old. Methods: The study group consisted of 40 volunteers aged 14–17 years practicing medium distance running for at least two years. The study lasted 12 days and followed a randomized, double-blind, placebo-controlled, parallel design. All subjects attended a familiarization session on day 0 before the test. The subjects were randomly divided into two groups: supplements and placebo group. The same training cycle protocol was used in both groups during the 12-day training period. Morning warm-up involved 10 min jogging at 60–75% of maximal heart rate and countermovement jump height measurement. Main training units were carried out for both groups with the same volume. Training load assessment (the daily session Rating of Perceived Exertion (s-RPE) method) method takes into consideration the intensity and the duration of the training session to calculate the “training load” (TL). Results: At the end of the training cycle, a significant (*p* = 0.002) decrease in the countermovement jump (CMJ) height was found in the placebo group when compared to the baseline. In the supplement group, there was no decrease in the countermovement jump height. Creatine kinase and lactate dehydrogenase concentration increased during the training days similarly in both groups and decreased on rest days. There were no differences between groups in enzymes concentration. The research results indicate that the supplement combination used in the supplements group prevented a reduction in the CMJ values. In contrast to the supplements group, in the placebo group, the CMJ changes were statistically significant: a noticeable (*p* = 0.002) decrease in CMJ was noted between the baseline measurement and the 6th measurement. The well-being of the subjects from both groups changed significantly during the training period, and the intergroup differences in the mood level were similar and not statistically significant. Conclusions: The results of this study indicate that the daily co-supplementation with calcium salt of β-hydroxy-β-methylbutyrate (7.5 g) and L-Arginine α-ketoglutarate (10 g) during training might help to prevent decline in jump performance. No influence on muscle damage markers or mood was shown.

## 1. Introduction

Pre-workout supplements, intended to be taken before workout, are popular class of dietary supplements among athletes. The prevalence of supplementation among athletes has been estimated at 37 to 89% [1].

β-hydroxy-β-methylbutyrate (HMB) is considered ergogenic aid for physically active people and is one of the best-examined compounds found in dietary supplements [2]. HMB is a derivative of leucine that has been extensively studied for post-workout recovery and protein metabolism including increase in muscle protein synthesis and decrease of muscle protein breakdown. This mechanism is crucial especially in case of injury, calorie restriction, immobilization or overtraining [3].

Several studies were focused on the effectiveness of Ca-HMB (calcium salt form) supplementation on muscular strength and body composition. Niessen et al. [4] showed that 1.5 g/day and 3.0 g/day of HMB taken daily by untrained males during a 3-week resistance training program, and the higher dose was more effective in one-repetition maximum (1-RM) testing protocol when compared to the placebo group. In addition, the total strength increased significantly by 13% (1.5 g HMB/d) and 18.4% (3.0 g HMB/day) when compared to placebo, and this dependency was dose dependent. Asadi et al. [5], during a 6-week study, also demonstrated the positive influence of HMB supplementation on strength and power performance compared to the placebo group.

Panton et al. [6] demonstrated that 4-week supplementation with 3 g of HMB per day during resistance training increased 1-RM strength (only in the HMB-group) in the upper-body exercises. No differences in the lower body strength between the placebo and the HMB groups were observed. In contrast, Wilson et al. [7] during their 12-week study involving 20 trained male subjects who were given 3 g of HMB per day observed strength increases in squat and bench press compared to the placebo group. In addition, some advantageous changes in body mass, fat free mass (FFM), body fat (BF), and quadriceps thickness were observed in the subjects supplemented with HMB.

Some researchers have investigated the effectiveness of HMB supplementation on anaerobic power (i.e., Wingate test) and countermovement jump (CMJ) performance [7,8]. The study conducted by Wilson et al. [7] showed an improvement in Wingate test results and CMJ performance, while Kreider et al. [8] did not observe any enhancement in 12 sets of 6 s cycle ergometer interval sprint tests.

Durkalec-Michalski et al. [9] reported that 12 weeks of supplementation with 3 g of HMB per day of highly-trained combat sports athletes, resulted in significant increase in the anaerobic peak power, average power, maximum speed, and post-exercise lactate concentration (after the Wingate test on a cycle ergometer) in comparison to placebo. Moreover, HMB supplementation increased the peak power output, average power, and maximum speed with a simultaneous reduction of the time needed to achieve peak power, compared to the placebo group.

L-Arginine (Arg) is a conditionally essential amino acid whose primary function is the participation in protein synthesis and ammonia detoxification [10]. It is also involved in several other functions related to its metabolism to biologically active particles, such as nitric oxide, creatine, agmatine, glutamine, ornithine, polyamines, or citrulline [10].

There is also some evidence that Arg supplementation could have a beneficial influence on anaerobic capacity. Yavuz et al. [11] examined the significance of a single dose of Arg (1.5 g/kg body weight) given to 9 males (national and international level wrestlers) after a 12-h (night) fast. The athletes performed an incremental bicycle ergometer test to exhaustion, measuring parameters: oxygen consumption, heart rate, and plasma lactate levels. The results showed that the significant difference was observed in time to exhaustion which was prolonged in the Arg group (1386.8 ± 69.8 s) when compared to placebo (1313 ± 90.8 s) (*p* < 0.05).

Bailey et al. [12] studied the effects of a 3-day supplementation with 6 g/day of Arg (dissolved in 500 mL of water) in 9 trained, healthy, active men 60 min before exercise on cycle ergometer. The participants were requested to complete 6 min cycling bouts with moderate intensity. The last cycle was continued until failure, and was used as a measure of exercise tolerance. There was significant increase in plasma nitrite and time to task failure in the Arg group when compared to the placebo group. In both groups, there was significantly lower oxygen consumption (VO_2_) during moderate-intensity cycle exercise. VO_2_ slow component amplitude was reduced during severe-intensity exercise, favoring the Arg group.

Mor et al. [13] observed no statistically significant difference before and after supplementation between the experimental (6 g of Arg) and placebo (6 g of wheat bran) groups consisting of 28 active male football players of amateur leagues during the 14-day research. Nevertheless, the post supplementation recovery and lactate levels showed more rapid reduction in the experimental group. Similar relationship was observed for the indicator of muscle injury—LDH enzyme levels were lower in the Arg group. All of this suggests that the supplementation with Arg supports lactate metabolism and increases muscle recovery by lowering the level of LDH enzymes when compared to the placebo group.

The use of Arg in post-workout recovery was the subject of research of McConell et al. [14], who submitted 9 endurance-trained males to a steady-state cycle ergometer exercise for 120 min. During the last 60 min of cycling, the subjects were given either placebo or Arg-HCl (30 g at 0.5 g/min) intravenously. Arg infusion significantly increased skeletal muscle glucose clearance, without increasing plasma insulin concentration. The authors suggested that Arg increased NO production, which then elevated muscle glucose uptake and might have helped muscle recovery.

Most studies on Arg indicate that it has no or little effect on anaerobic capacity. However, some experiments show directional activity, mainly due to the presence of nitric oxide (NO) derived from Arg, which seems to protect muscle tissue from damage and aids the recovery. Similar results are associated with HMB and the enhancement of the sarcolemma integrity properties via higher availability of cytosolic cholesterol [14], inhibiting protein degradation [15], decreasing cell apoptosis [16], increasing protein synthesis (mTOR pathway) [14], stimulating the growth hormone (GH)-insulin-like growth factor-1 (IGF-1) axis and enhancing muscle stem cells proliferation and differentiation [14].

Campbell et al. [17] reported significant increases in the 1-RM strength and anaerobic power (Wingate test) in 35 resistance-trained healthy males after supplementation of 12 g of L-Arginine α-ketoglutarate (AAKG); 6 g of Arg) per day for eight weeks. The authors observed a significant increase in the peak power in the AAKG group in comparison to the placebo group. α-ketoglutarate is a compound that plays an important role in metabolism and participates in numerus biochemical reactions including energy production and amino acids synthesis. This compound is often found in dietary supplements, especially in combination with a L-Arginine [18]. As literature shows, AAKG supplementation is safe and well tolerated and it also is a frequently chosen type of dietary supplement for athletes [17]. There is a significant number of research on L-Arginine administrated in the form of free amino acid. Some authors emphasize the need for research concerning different compounds of L-Arginine, especially those concentrating on human health and physical performance [19].

It would be interesting whether these two popular substances (HMB and AAKG) used simultaneously show an additive effect, especially during intense anaerobic workout. This combination of supplements has not been previously studied.

The purpose of this study was to check whether two popular supplements, HMB and AAKG, would have beneficial influence on blood biochemical parameters (creatine kinase—CK and lactate dehydrogenase—LDH) and values of lower limb strength and power. We also wanted to check the possible influence on subjective perception of fatigue and well-being. The study group was young, middle distance runners during a 12-day intensive preparatory camp. Based on the previously mentioned molecular mechanism of action of L-Arginine and HMB, we hypothesized that the combination of these two compounds can have an additive effect for examined parameters. Our goal was to study the possible effect of HMB and AAKG taken simultaneously.

## 2. Materials and Methods

### 2.1. Study Design

Following an explanation of all procedures, risks, and benefits associated with the study, each subject gave his written consent before participating in the study. In addition, the statement regarding the obtainment of consent from a minor’s parent or guardian was required. The study was approved by the Ethical Committee of the Academy of Physical Education in Katowice (Katowice, Poland) and conformed to the ethical requirements of the 1975 Helsinki Declaration. The subjects were also required to refrain from taking any nutritional supplements or ergogenic aids for the two weeks preceding the study. They were also asked to not to take any additional supplements during the duration of the study.

The study followed a randomized, double-blind, placebo-controlled design. All subjects attended a familiarization session on day 0 before the test commenced. The subjects were also requested not to eat or drink for 2 h before each training session. During the familiarization session, the participants were shown and explained the rules for completing a well-being questionnaire and a session Rating of Perceived Exertion (sRPE) protocol; they were also informed about planned tests during the entire 12-day experiment (Table 1) as well as their order on day 1 (Table 2; daily schedule of meals, supplementation, and frequency of training as well as samples and data collection) and during the entire 12-day period (Table 1). During the familiarization session, baseline values for CMJ were established and the questionnaire of well-being was filled.

### 2.2. Subjects

The study group consisted of 40 volunteers (men and women) aged 14–17 (juniors and younger juniors) who have been practicing medium distance running for at least two years; the subjects were randomly assigned to the placebo (PL) or supplements (SUP) group. The study was completed by 15 subjects from the placebo group, including 7 men (15.1 ± 1.2 y; 64.6 ± 7.8 kg; 171.4 ± 6.6 cm) and 8 women (15.0 ± 1.2 y; 52.6 ± 5.5 kg; 158.9 ± 5.8 cm). In the supplementation group (SUP), 19 subjects completed the study, including 7 men (15.0 ± 1.0 y; 66.0 ± 3.9 kg; 173.0 ± 7.3 cm) and 12 women (15.3 ± 1.3 y; 50.6 ± 3.8 kg; 156.1 ± 7.2 cm). Six participants were excluded from the study due to taking capsules inconsistently with the protocol.

### 2.3. Supplementation

The participants were randomly divided into two groups. The placebo group (PL) was administered hard, gelatin capsules with microcrystalline cellulose. In contrast, the supplements group (SUP) was given a combination of two supplements which are commercially marketed as HMB 1250 Mega Caps^®^ (Olimp Laboratories, Pustynia, Poland; HMB) of which one capsule contains 1250 mg of calcium salt of β-hydroxy-β-methylbutyrate (Ca-HMB) and AAKG 1250 Extreme Mega Caps^®^ (Olimp Laboratories, Pustynia, Poland; AAKG) of which one capsule contains 1250 mg of L-Arginine α-ketoglutarate which equals 813 mg of L-Arginine. Each participant took them three times a day approximately 15 min before early morning warm-up and then 60 min before the first and the second training. A set dose of the supplements or placebo was administrated as described in Table 2. Each dose was taken with 200 mL of still water. No subjects reported any adverse events or side effects following the ingestion of the supplement or placebo.

### 2.4. Training

#### 2.4.1. Early Morning Warm-Up

Standard warm-up started with 10 min jogging at 60–75% of maximal heart rate. After this task, the participants performed various dynamic exercises (arm swing, internal/external leg rotation, hip flexion/extension/abduction/adduction/hip rotation, knee rotation, and ankle rotation) followed by 15 min jogging.

#### 2.4.2. Main Training Units

The training cycle comprised of general preparation units, including off-road running games, as well as specific units. Each training unit consisted of four parts: introduction (presenting the training goal and tasks, dividing into smaller training groups: 3–5 min), initial (warm-up jogging, dynamic stretching for 20–25 min), main (achieving the fundamental training goal for 75–80 min), and final (easy jog, flexibility exercises for 15 min).

The training units were identically arranged for both groups and had the same volume. Each day, the selected training unit (morning or afternoon) was carried out with a greater load. Session RPE was used to describe the training load of each workout, as presented below. Similar levels of session RPE were observed in the athletes in both groups (SUP and PL). During the boot camp, both groups (PL and SUP) trained 36 times in total. The total training time was 54 h per athlete from each group. The seventh day of the camp was partially a rest period—only one, low-intensity training took place then.

#### 2.4.3. Fatigue and Well-Being Assessment

**Figure A1:**
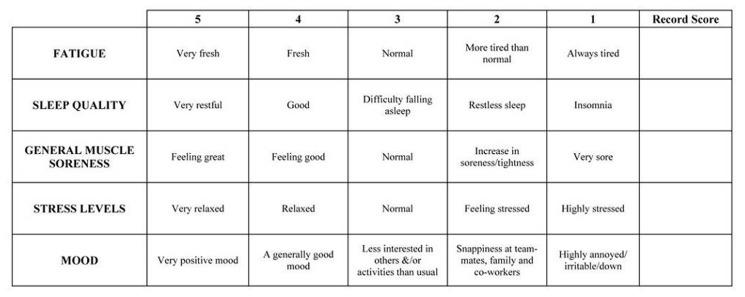
The Fatigue and Well-Being Assessment pattern.

The day before the camp began, as well as every morning during its duration, a psychometric survey was carried out before the start [20]. The survey comprised of five questions related to perceived fatigue, sleep quality, general muscle soreness, stress levels and mood; each question was scored on a 5-point scale (with 1 and 5 representing poor and very good wellness ratings, respectively) [21]. Using the questionnaire, the participants made a subjective assessment of overall fatigue and well-being. The Fatigue and Well-Being Assessment pattern was placed in Appendix A (as a Figure A1) just above the References.

#### 2.4.4. Training Load Assessment

The session-RPE (sRPE) method takes into consideration the intensity and the duration of the training session to calculate the “training load” (TL) [22]. The training load (arbitrary units) was calculated for all subjects as total training duration (min) × session rating of perceived exertion (RPE, CR10 modified Borg’s scale), collected within 10 min of completing each training session [23].

All athletes were familiarized with the CR-10 scale according to standard procedures [24] before beginning to collect reliable measurements. Immediately after the end of each training unit, each participant reported the subjective value of the session RPE, which was recorded by the coach. The mean value of the two daily sRPE values was used for statistical calculations.

#### 2.4.5. Countermovement Jump

The countermovement jump measurements were taken using the Optojump Next system (Microgate, Bolzano, Italy)—an optical measuring system used in time-based tests. For example, the vertical jump height is calculated from the value of this measurement. CMJ is used as an indirect measurement of lower limb power. The measurement was taken daily after the morning warm-up. The starting position was standing upright with the arms placed on the hips to prevent them from swinging. The athletes were instructed to begin with the eccentric phase—make a preliminary downward movement by flexing at their knees and hips to the semi-squatting position and immediately perform the highest jump possible (concentric phase). The landing was allowed with normal flexion and the standing still phase in the neutral position was obligatory for 1–2 s. Bending the lower limbs before performing the jump causes initial stretching of the muscles, consequently more work is done in the concentric phase. Each of the subjects made three countermovement jumps with 1-min rest between them. The average value of the three jumps was used for calculations.

### 2.5. Biochemical Analyses

Blood samples were taken by qualified medical personnel and used to assess the concentration of the creatine kinase (CK) and lactate dehydrogenase (LDH) enzymes as the markers of the level of muscle tissue damage. CK and LDH tests were performed in a laboratory that validated methods for the determination of these particles using biochemical tests (i-STAT^®^ CK-MB (Abbott, Chicago, IL, USA) (biological material—blood serum, analytical sensitivity ≤ 0.1 ng/mL), and Lactate Dehydrogenase (LDH) ELISA Kit (MyBioSource, San Diego, CA, USA) (biological material—blood serum, analytical sensitivity 5.0 U/L). Blood samples were taken six times (every 48 h) from a vein in the arm (4 mL of blood) in the morning—fasting state and before morning warm-up. The participants were instructed to rest for a few minutes in a sitting or lying position and to avoid physical activity immediately before blood samples were to be taken. The first blood samples were taken in the morning on the first training day (baseline). The results from 6 measurement points were collected.

### 2.6. Statistical Analysis

The changes in the analyzed indicators and intergroup differences in the level of the analyzed indicators were determined using analysis of variance (ANOVA) with repeated measurements the supplementation could possibly have different effect on CMJ height in women and men. We performed multivariate analysis of variance for factors: group and sex with repeated measurements. Data distribution was checked using the Shapiro–Wilk test. The homogeneity of variance within the groups was tested with the Levene’s test. In the case of significant changes for the main effect (*p* < 0.05), post hoc analysis was performed using the Tukey test. Statistical analysis was conducted in the Statistica program (Statsoft, Tulsa, OK, USA). The data in the charts presented are an average with a confidence interval of 0.95. Any changes in the indicators were considered significant when *p* < 0.05.

## 3. Results

**Figure 1:**
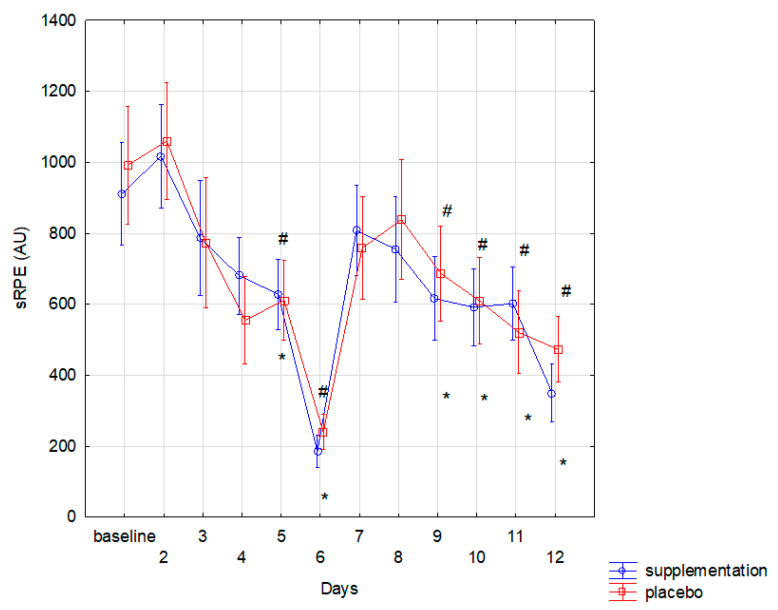
The changes of session Rating of Perceived Exertion (sRPE) during training in group with supplementation and with placebo (* significant change (*p* < 0.05) to baseline in placebo group; # significant change (*p* < 0.05) to baseline in supplement group).

No subjects reported any adverse events or side effects following the ingestion of the supplement or placebo. The athletes from both groups had comparable training load. The sRPE reported in both groups was similar (f = 0.07, *p* < 0.001). sRPE result tended to decline in subsequent days in both groups. Compared to the baseline, significantly (*p* < 0.05) lower sRPE was noted in both groups on the 5th, 6th, 9th, 10th, 11th, and 12th day (Figure 1).

**Figure 2:**
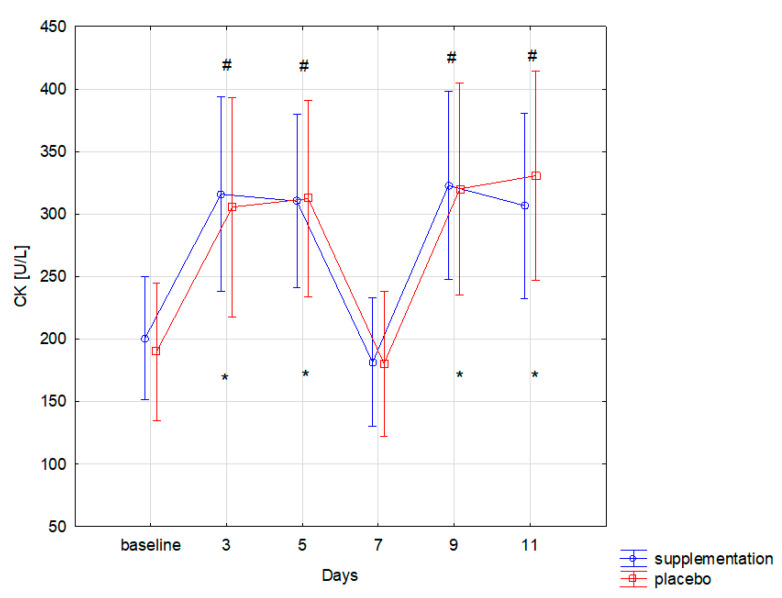
Creatine kinase (CK) concentration in subsequent days in supplements (SUP) and placebo (PL) groups (* significant change (*p* < 0.05) to baseline in PL group; # significant change (*p* < 0.05) to baseline in SUP group).

**Figure 3:**
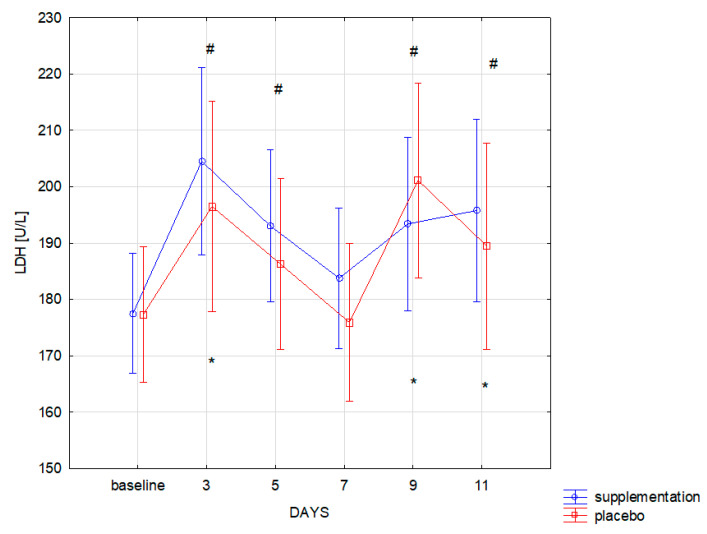
Lactate dehydrogenase (LDH) concentration in subsequent days in SUP and PL groups (* significant change (*p* < 0.05) to baseline in PL group; # significant change (*p* < 0.05) to baseline in SUP group).

The training load in similar muscle damage in both groups, as expressed by the CK level (f = 0.00, *p* = 0.99). Changes in CK concentration during the 12-day training period were statistically significant (f = 38.09, *p* ≤ 0.001). Significantly higher concentration of CK, compared to the baseline, was recorded in both groups on the 3rd, 5th, 9th, and 11th day of training camp (Figure 2). Similar changes were also observed for LDH: group differences were statistically insignificant (f = 0.13, *p* = 0.71). Changes during follow-up were statistically significant (f = 18.01, *p* < 0.001). Significant (*p* < 0.05) increase in LDH concentration compared to the baseline was recorded on the 3rd, 9th, and 11th day of training in both groups (Figure 3).

**Figure 4:**
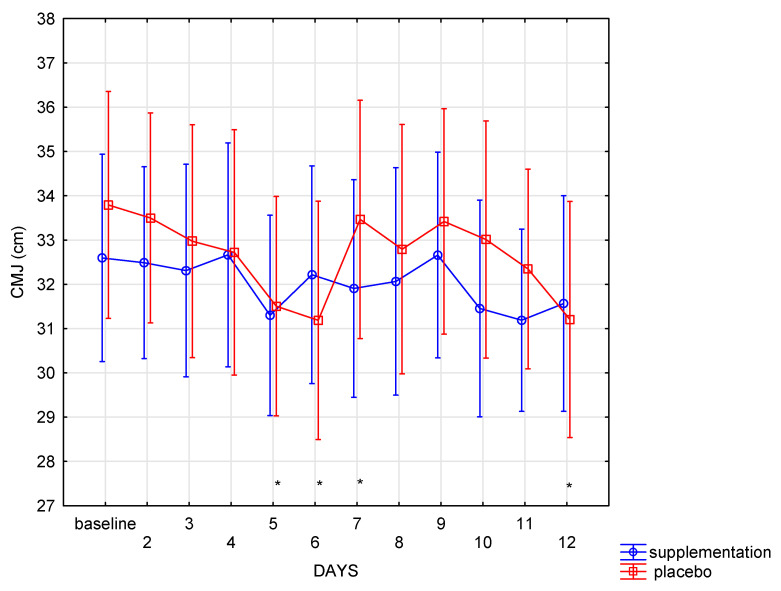
Countermovement jump (CMJ) height in subsequent days in SUP and PL groups (* significant change (*p* < 0.05) to baseline in PL group).

The changes in CMJ height in the analyzed period were particularly interesting—there were no differences between SUP and PL groups in CMJ (f = 0.14, *p* = 0.70). However, in both groups, the changes in the CMJ height proceeded differently (interaction group × days: f = 1.92, *p* = 0.04). Post hoc analysis showed that in the supplement group, no significant differences (*p* > 0.05) were noted between subsequent days. On the last day of training, the CMJ height was close to the baseline. In contrast, in the placebo group, the CMJ height changes were statistically significant: decrease in CMJ height (*p* = 0.002) was noted between the baseline and the 6th day. At the same time, a considerable increase in CMJ height (*p* = 0.02) between the 6th and the 7th measurement was also recorded. On the last day, a significant (*p* = 0.002) decrease in the CMJ height was observed in the placebo group compared to the baseline measurement. There were no such changes registered in the SUP group (Figure 4).

We did not observe any different effect of supplementation on CMJ in women and men (f = 2.2; *p* = 0.15) and no interactions between factors (group × sex: f = 0.88, *p* = 0.35; days × sex: f = 0.61; *p* = 0.82; days × sex × group: f = 0.69, *p* = 0.74) were noted.

**Figure 5:**
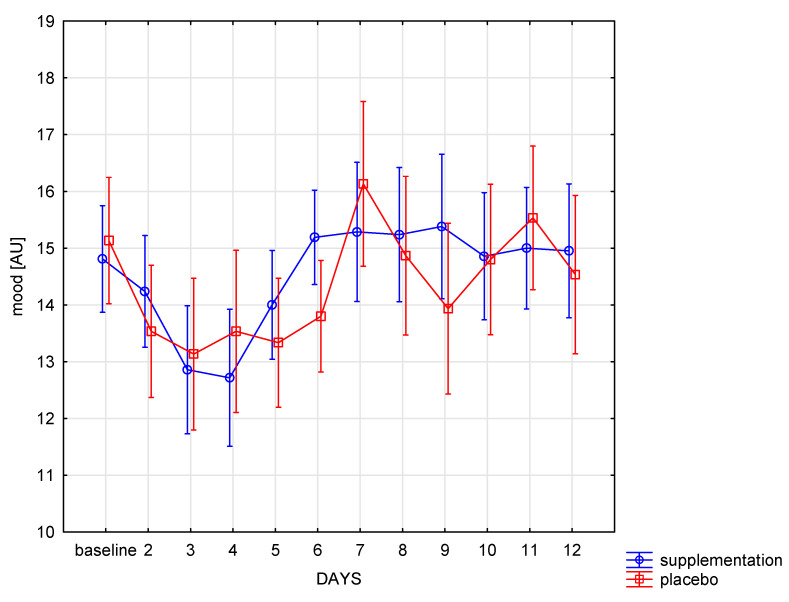
The point sum in sRPE scale in subsequent days in SUP and PL groups.

The well-being changed significantly during the training period in both groups (f = 6.62, *p* < 0.001), and the intergroup mood differences were similar (f = 0.12, *p* = 0.73). Significant (*p* < 0.05) improvement in well-being in the SUP group was noted between the 3rd day, 6th, 7th, 8th, and 9th day and between 4 and subsequent days of training. Afterwards, the well-being did not change until the end of the camp. In the placebo group, significant (*p* < 0.05) improvement in well-being was shown only after day 7 compared to the previous days (days 2–6) (Figure 5). In the following days, no significant changes were observed in both groups.

There were no significant interactions between the analyzed factors (group × days) (sRPE: f = 1.13, *p* = 0.33; CK: f = 0.35, *p* = 0.87; LDH: f = 2.04, p-0.07; mood: f = 1.39, *p* = 0.17).

## 4. Discussion

The purpose of this study was to check whether the intensive use of two popular supplements, HMB and AAKG, would reduce muscle damage, increase lower limb muscle power, and affect the subjective perception of the effort severity and well-being in the group of young medium distance runners. Significant intergroup differences in lower limb power are the most important discovery of our research. In the supplement group, no significant fluctuations in muscle power were observed during the follow-up. In the placebo group, power changes were statistically significant, and at the end of the boot camp unquestionably lower than before the boot camp started. Such power changes can adversely affect the implementation of the training plan and force the change of the training load. HMB and AAKG supplementation seemed to help to maintain stable power level, which should be considered beneficial. The placebo group showed a decrease in the CMJ height in the first days. This outcome could have been a result of increased fatigue due to the implementation of subsequent, intensive units of running training. At the same time, no such trend was observed in the SUP group. Although there were no statistical differences in the training program and the subjective assessment of the training load (similar sRPE), a statistically significant decrease in CMJ was shown only in the placebo group. Reduction in the CMJ height was not observed in the SUP group, in which CMJ performance remained relatively stable. The research results indicate that, the combination of the two supplements prevented the deterioration of the CMJ results, which were observed in the placebo group. It should be emphasized that it was only after the camp ended that power supercompensation was to be expected. Research to date [25,26] indicates that during the training period, the CMJ height may either decrease or not undergo significant changes. The authors suggest that this may be a result of fatigue accumulation, what could be possibly prevented by co-supplementation of HMB and AAKG.

The chemical form of Arg used in the study, AAKG, as well as the calcium form of HMB, are often used in general foods, dietary supplements, and foods for special medical purposes. These compounds have long history of use and excellent safety profile.

To this date, a few possible mechanisms of HMB activity in the human body were proposed. The positive effects of HMB supplementation include enhancing sarcolemma integrity via higher availability of cytosolic cholesterol [14], inhibiting protein degradation (ubiquitin pathway) [15], decreasing cell apoptosis [16], increasing protein synthesis (mTOR pathway) [14,26], stimulating the growth hormone (GH)-insulin-like growth factor-1 (IGF-1) axis, and enhancing muscle stem cells proliferation and differentiation [16].

Arg-based products are used mainly before training, due to the potential mechanism of action of nitric oxide (NO) derived from the amino acid. NO in muscles is responsible for vascular smooth muscle relaxation, thus improving the supply of oxygen and nutrients to the working muscles, as well as for better drainage of anaerobic energy metabolism [27]. In addition, nitric oxide is involved in generating the contraction strength of working muscles by affecting the excitatory-contraction coupling. It is also indicated that Arg supplementation reduces the aerobic cost of physical activity [28]. This effect can also be caused by a decrease in the concentration of lactate and ammonia in the bloodstream [29] or an increase in the glucose absorption efficiency by muscle cells [14] to which Arg also contributes.

Co-administration of AAKG [12] and Ca-HMB [14] was also expected to cause a reduction in the muscle cell damage markers and thus indirectly faster post-workout regeneration were expected. The effect of supplementation on lower limb power and well-being and subjective assessment of the intensity of each training unit declared in the survey were also examined. The effects of co-administration of only AAKG and HMB have not yet been described in the literature. However, adding L-glutamine to the mixture (3 g HMB, 14 g L-Arginine, 14 g L-glutamine) administered for six months to healthy elderly helped to increase the lean muscle mass [30]. An important finding is that no statistically significant differences in the level of muscle cell damage markers (CK, LDH) were observed between SUP and PL groups. This could be caused, i.e., by individually variable myocyte stability and the ability to metabolize CK by the athletes in both groups. In addition, Kupiers [31] concluded that in some cases, serum CK might not directly reflect the extent of muscle cell damage. This correlation depends on the level of sex hormones and individual variability, for example, on FFM (fat-free mass) [31]. The possible reason for the lack of any changes in the level of muscle cell damage markers (CK, LDH) could be a short duration of the study and supplementation. Basing on the research by Niessen et al. [4], Ca-HMB most effectively inhibits protein degradation in the first two weeks of use, while a significant reduction in serum CK activity occurs only in the third week of supplementation. The decrease in CK and LDH values in point 4 of Figure 2 and Figure 3 was probably due to the fact that it reflected a day without training, and therefore no additional muscle damage was induced by intense training units.

In this study, AAKG and Ca-HMB were co-administered in the SUP group. A total of 6.5 g derived from 10 g AAKG (8 capsules) was the set optimal daily dose of Arg. The dosage of 6 g increased muscle blood volume during recovery from sets of resistance exercise [32]. The highest absorption of Arg (about 70%) is observed at the dose of 6g [33] and further dose increase does not change its uptake. A dose of 6 g of Arg taken in fasting state may increase the concentration of this amino acid in the blood by more than 330% within 1 h. It is recommended to take products based on Arg approximately 60 min before planned exercise, especially since the half-life tested at the optimal dose is about 80 min [32].

Based on the scientific data, it has been observed that the effect of HMB is dose-dependent [34,35]. The most common HMB dose in scientific research is 1.5 g/d, 3 g/d, or exceptionally 6 g/d [35]. We decided to increase the standard saving dose of 3g Ca-HMB 2.5-fold to 7.5 g Ca-HMB due to the short duration of the experiment (12 days), and what is more, literature shows the effect and potency of HMB are dose-dependent [3]. Although the literature data describe the fractional synthesis (FSR) representing MPS of myofibrillar proteins was increased after approximately 150 min of HMB administration by approximately 70% above the baseline level [36]. Nevertheless, the literature states that the minimum period of HMB supplementation, when the first physiological effects are noticeable occurs in a 3-week study with untrained participants [37], which under the existing experimental conditions (12-days) was impossible to achieve, which of course does not exclude further research in this direction and it should be checked how extended, at least to 6 or even 8 to 12 weeks. We assume that simultaneous supplementation of HMB and AAKG affect the parameters tested in this study. We do not want to miss the effect of HMB during the study, so we want to achieve a much faster saturation of the body with HMB and AAKG, higher than commonly used in research dosages. In addition to that, based on the results of Baxter et al. [38], the no-observed-adverse-effect level (NOAEL) was considered to be 5% of Ca-HMB mixed with diet (3.49 g/kg BW for males and 4.16 g/kg BW for females).

It is assumed that the optimal model for minimizing the damage to the trained muscle is a supplementation protocol of 1 g of Ca-HMB taken three times a day with meals. For athletes, to shorten post-exercise recovery time, it is recommended to consume 3 g Ca-HMB 60 min before exercise [34,35]. A study on the effect of HMB supplementation on the level of muscle tissue damage markers after endurance exercise (20 km cross-country run) was conducted. The participants were randomly assigned to placebo or supplemented group (3 g HMB per day for six weeks). At the end of the study, a 20 km run test was carried out, which showed statistically significant lower levels of both CK and LDH activity in the athletes regularly taking HMB. At the same time, in both groups, the peak CK level was recorded 24 h after the 20 km run. [39]. No similar results were obtained in our study.

The study protocol it was decided to use 6 g HMB to check if this dosage is sufficient to positively influence the analyzed parameters in the limited period of the 12-day training camp. The fact that the plasma HMB half-life is 2.5 h when administered as calcium salt (Ca-HMB) and that taking 3 g of Ca-HMB would result in the peak concentration of pure HMB in the bloodstream after 1 h [40] contributed to Ca-HMB and AAKG being co-administered three times a day about 1 h before physical activity. The use of higher doses than those most commonly found in the literature was aimed at optimizing the rate at which the expected effects would appear (reduced level of the muscle cell damage markers, counteracted decrease in lower limb power, improved well-being, and lowered subjective feeling of effort intensity (sRPE).

## 5. Conclusions

Training with or without supplementation induced muscle damage compared to baseline, and decreased jump performance only in the control group but not in the Ca-HMB + AAKG group. It suggests that Ca-HMB + AAKG might prevent exercise-induced declines in performance, without changing muscle damage or mood.

## 6. The Limitations of the Study

In this study, we focused on the effects of Ca-HMB + AAKG on jump performance. We did not evaluate the separate impact of Ca-HMB or AAKG on jump performance. In our opinion, it is necessary also to assess the effects of co-supplementation with HMB and AAKG after the end of the camp (training period), when after a period of complete rest, muscle power can significantly increase (supercompensation). Perhaps then other supplementation effects could also be observed.

## Figures and Tables

**Table 1:** Daily schedule of tests performed during the 12 days of the experiment.

	Familiarization/Baseline Values						
	day 0	day 1	day 2	day 3	day 4	day 5	day 6
Blood samples		+		+		+	
CMJ	+	+	+	+	+	+	+
well-being questionnaire	+	+	+	+	+	+	+
sessionRPE		+	+	+	+	+	+
		day 7	day 8	day 9	day 10	day 11	day 12
Blood samples		+		+		+	
CMJ		+	+	+	+	+	+
well-being questionnaire		+	+	+	+	+	+
sessionRPE		+	+	+	+	+	+

**Table 2:** Daily regimen during the 12-day study period.

Time	Activity	Supplements Group (SUP)	Placebo Group (PL)
6:00–6:15	Blood sample collections	+	+
	SUP/PL	2 caps AAKG + 2 caps Ca-HMB	4 caps placebo
	Well-being questionnaire	+	+
6:15–6:45	Morning warm-up	+	+
6:45–7:00	Counter movement jump test	+	+
7:00–8:00	Breakfast		
9:00	SUP/PL	3 caps AAKG + 2 caps Ca-HMB	5 caps placebo
10:00–12:00	Training 1	+	+
	sessionRPE 1	+	+
13:00–14:00	Lunch		
15:00	SUP/PL	3 caps AAKG + 2 caps Ca-HMB	5 caps placebo
16:00–18:00	Training 2	+	+
	sessionRPE 2	+	+
19:00–20:00	Dinner		

## Data Availability

The datasets used and/or analyzed during the current study are available from the corresponding author on reasonable request.

## Appendix A

The well-being review sheet that was completed during the study [41].
